# Curcumin sensitizes TRAIL-resistant xenografts: molecular mechanisms of apoptosis, metastasis and angiogenesis

**DOI:** 10.1186/1476-4598-7-16

**Published:** 2008-01-29

**Authors:** Sharmila Shankar, Suthakar Ganapathy, Qinghe Chen, Rakesh K Srivastava

**Affiliations:** 1 Department of Biochemistry, University of Texas Health Science Center at Tyler, Tyler, Texas, 75703, USA

## Abstract

**Background:**

We have recently shown that curcumin (a diferuloylmethane, the yellow pigment in turmeric) enhances apoptosis-inducing potential of TRAIL in prostate cancer PC-3 cells, and sensitizes TRAIL-resistant LNCaP cells *in vitro *through multiple mechanisms. The objectives of this study were to investigate the molecular mechanisms by which curcumin sensitized TRAIL-resistant LNCaP xenografts *in vivo*.

**Methods:**

Prostate cancer TRAIL-resistant LNCaP cells were implanted in Balb c nude mice to examine the effects of curcumin and/or TRAIL on tumor growth and genes related to apoptosis, metastasis and angiogenesis.

**Results:**

Curcumin inhibited growth of LNCaP xenografts in nude mice by inducing apoptosis (TUNEL staining) and inhibiting proliferation (PCNA and Ki67 staining), and sensitized these tumors to undergo apoptosis by TRAIL. In xenogrfated tumors, curcumin upregulated the expression of TRAIL-R1/DR4, TRAIL-R2/DR5, Bax, Bak, p21^/WAF1^, and p27^/KIP1^, and inhibited the activation of NFκB and its gene products such as cyclin D1, VEGF, uPA, MMP-2, MMP-9, Bcl-2 and Bcl-X_L_. The regulation of death receptors and members of Bcl-2 family, and inactivation of NFκB may sensitize TRAIL-resistant LNCaP xenografts. Curcumin also inhibited number of blood vessels in tumors, and circulating endothelial growth factor receptor 2-positive endothelial cells in mice.

**Conclusion:**

The ability of curcumin to inhibit tumor growth, metastasis and angiogenesis, and enhance the therapeutic potential of TRAIL suggests that curcumin alone or in combination with TRAIL can be used for prostate cancer prevention and/or therapy.

## Introduction

The process of malignant transformation involves the sequential acquisition of a number of genetic and epigenetic alterations as a result of increasing genomic instability caused by defects in checkpoint controls [1,2]. These alterations allow cancer cells to acquire the capabilities to become self-sufficient in mitogenic signals, deregulate the control of cell cycle, escape from apoptosis, and obtain unlimited replication potential [3-5]. Within a growing tumor mass, the genetic changes during tumor progression also enable cancer cells to gain the ability to induce angiogenesis, invade neighboring tissues, and metastasize to distinct organs [6]. The new chemopreventive agents or therapeutic strategies that inhibit angiogenesis, metastasis and invasion can be considered for future clinical development.

Epidemiological data have demonstrated that curcumin is safe, non-toxic, and has long lasting beneficial effects on human health. Curcumin [1,7-bis(4-hydroxy-3-methoxyphenyl)-1,6-hepatadiene-3,5-dione; diferulolylmethane], a major constituent of the yellow spice turmeric, is derived from the rhizomes of *Curcuma spp*. [7]. It possesses antitumor, anti-inflammatory and anti-oxidant properties [7,8]. In addition, curcumin has been shown to inhibit tumor metastasis, invasion and angiogenesis [9-12]. We have recently shown that Bax and Bak genes completely inhibited curcumin-induced apoptosis in Bax^-/- ^and Bax ^-/- ^mouse embryonic fibroblasts [13], and curcumin induced apoptosis in prostate cancer cells by inhibiting Akt activity upstream of mitochondria [14]. These data suggest that curcumin regulates multiple signaling pathways and possesses several therapeutic benefits.

Nuclear factor (NFκB) is a dimeric DNA binding protein consisting of members of the NFκB/Rel family [15]. Its expression is ubiquitous in mammalian cells. Normally, NFκB resides in the cytoplasm in an inactive form in association with inhibitory proteins. These inhibitory proteins, which belong to a family of proteins named inhibitor of NFκB [15], prevent NFκB nuclear translocation by masking the NFκB nuclear localization signal and thus, inhibit NFκB DNA binding and transactivational function [15,16]. Various stimuli activate a large number of distinct signaling pathways that eventually result in the phosphorylation of inhibitor of NFκB and its subsequent degradation by the proteasome or its dissociation from NFκB without additional degradation [15-17]. The released NFκB then translocates to the nucleus and binds to κB DNA motifs to initiate gene transcription. The putative target genes of NFκB are involved in immune and inflammatory responses, and in the control of cell proliferation, apoptosis, metastasis and angiogenesis [15,16]. Tumor cells usually express high levels of constitutively active NFκB [16,18]. Furthermore, curcumin inhibited NFκB activity in cancer cells [9,19] and sensitized cancer cells to chemotherapy and radiotherapy [20-25].

TNF-related apoptosis-inducing ligand (TRAIL) binds to TRAIL-R1/DR4 and TRAIL-R2/DR5. TRAIL induces apoptosis in cancer cells of various origins [26-30]. Data on experimental animals and primates led us to believe that TRAIL has great promise as a selective anticancer agent [27,28,31]. We have recently demonstrated that TRAIL induces apoptosis in several prostate cancer cells lines, but it was ineffective in inducing apoptosis in LNCaP cells [27,28,32]. Furthermore, curcumin sensitizes TRAIL-resistant prostate cancer cells to growth inhibition by TRAIL *in vitro *[33-35]. However, the ability of curcumin to sensitize TRAIL-resistant prostate cancer cells *in vivo *has not yet been demonstrated.

The purpose of our studies was to investigate the molecular mechanisms by which curcumin sensitized TRAIL-resistant prostate cancer cells *in vivo*. Our results indicated that curcumin inhibited growth, metastasis, and angiogenesis of TRAIL-resistant LNCaP xenografts in nude mice through regulation of NFκB and its gene products, and sensitized these xenografts to TRAIL treatment. Thus, curcumin can be used alone or combined with TRAIL for prostate cancer prevention and/or therapy.

## Results

### Curcumin sensitizes TRAIL-resistant tumor cells *in vivo*

**Figure 1:**
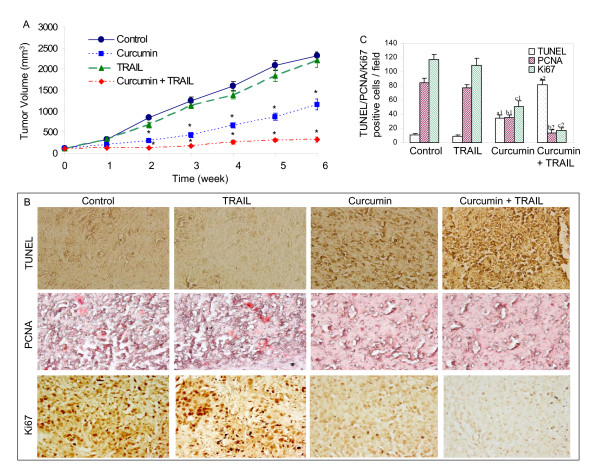
Curcumin sensitizes TRAIL-resistant LNCaP xenografts. (A), LNCaP cells were injected into the right flanks of Balb c nude mice. After tumor formation (about 100 mm^3^), mice were treated with saline, curcumin (30 mg/kg, three days per week), TRAIL (15 mg/kg, four times during first three weeks) or curcumin and TRAIL. Tumor volume was calculated weekly. Data represent mean ± SE. (B), Effects of curcumin and/TRAIL on apoptosis (TUNEL staining) and cell proliferation (PCNA and Ki67 staining). Immunohistochemistry was performed to measure apoptosis (TUNEL assay), and expression of PCNA and Ki67 in tumor tissues derived from control and treated mice on week 6. (C), Quantification of TUNEL, PCNA and Ki67 positive tumor cells. Tumor slides of different treatment groups were visualized under microscope, and TUNEL, PCNA and Ki67 positive cells were quantified. Data represent mean ± SE. a1, a2; b1, b2 and c1, c2 are significantly different from their respective controls or TRAIL treated group (P < 0.05).

We have recently shown that curcumin sensitizes TRAIL-resistant prostate cancer LNCaP cells *in vitro *[35]. Therefore in the present study, we examined the ability of curcumin to sensitize TRAIL-resistant LNCaP cells *in vivo*. LNCaP cells were xenografted in Balb c nude mice. After tumor formation, these mice were treated with curcumin, TRAIL, or curcumin plus TRAIL for 6 weeks to examine their effects on tumor growth and markers of proliferation, apoptosis, metastasis, invasion and angiogenesis. While TRAIL was ineffective in inhibiting the tumor growth, curcumin inhibited the growth of LNCaP xenografts in nude mice (Fig. 1A). Interestingly, curcumin sensitized TRAIL-resistant xenografts in nude mice, which was evident in reduction in tumor volume starting from week 2.

Since tumor growth is determined by the balance of cell proliferation and apoptosis, mechanisms that promote cell survival or prevent apoptosis of cancer cells would favor the establishment of tumor colonies. To test this, we examined the extent of cellular apoptosis in those tumors. Examination of tumor tissues by TUNEL assay revealed that curcumin alone induced apoptosis (Fig. 1B and 1C). A very few apoptotic cells were found in the highly vascularized tumors derived from control mice. TRAIL treatment of mice had no significant effect on apoptosis compared to control mice. On the other hand, treatment of mice with a combination of curcumin and TRAIL significantly showed more apoptosis than that of mice treated with curcumin alone. Immunohistochemical studies also demonstrated that curcumin inhibited cell proliferation (PCNA and Ki67 staining) in xenogrfated tumors (Fig. 1B and 1C). TRAIL had no effect on tumor cell proliferation because the staining of PCNA and Ki67 was not affected. The combination of curcumin plus TRAIL demonstrated slightly less tumor cell proliferation than curcumin alone. These data are in agreement with our recently published *in vitro *data where curcumin sensitized TRAIL-resistant LNCaP cells [35].

### *In vivo *regulation of Bcl-2 family members and death receptors by curcumin and/or TRAIL

**Figure 2:**
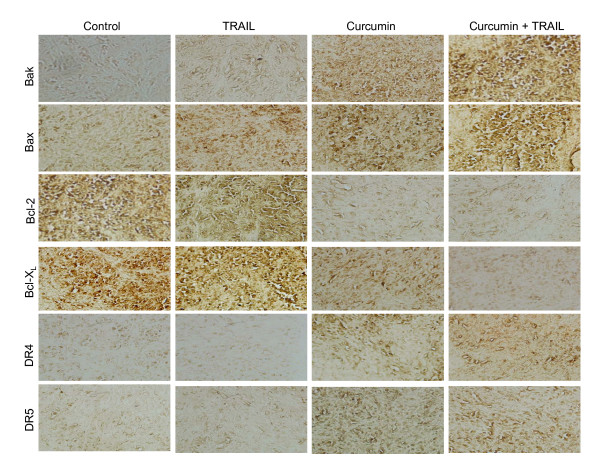
Immunohistochemical examination of Bcl-2 family members and death receptors. Immunohistochemistry was performed to measure the expression of Bak, Bax, Bcl-2, Bcl-X_L_, TRAIL-R1/DR4 and TRAIL-R2/DR5 in tumor tissues derived from control and/or treated mice on week 6.

Since curcumin sensitized TRAIL-resistant cells *in vivo*, we sought to examine the molecular mechanisms by which curcumin sensitized TRAIL-resistant LNCaP cells. We next examined the effects of curcumin and/or TRAIL on the expression of Bcl-2 family members (Bax, Bak, Bcl-2, and Bcl-X_L_) and death receptors (TRAIL-R1/DR4 and TRAIL-R2/DR5) by immunohistochemistry in tumor tissues derived from *in vivo *experiment (Fig. 2). Treatment of mice with curcumin enhanced the expression of Bax, Bak, DR4 and DR5, and inhibited the expression of antiapoptotic Bcl-2 and Bcl-X_L _proteins. TRAIL has no significant effect on the expression of Bak, Bax, Bcl-2, Bcl-X_L_, DR4 and DR5. On the other hand, treatment of mice with a combination of curcumin and TRAIL significantly showed more expression of Bax, Bak, DR4 and DR5, and less expression of Bcl-2 and Bcl-X_L _proteins than that of mice treated with curcumin alone or TRAIL alone.

**Figure 3:**
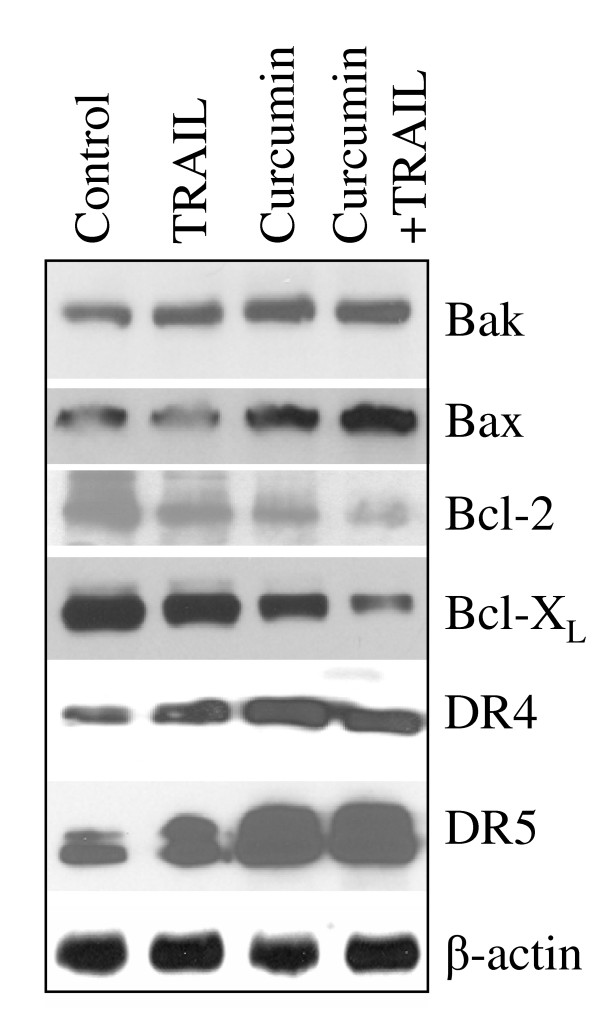
Western blot analysis of Bcl-2 family members and death receptors. Protein expression of Bak, Bax, Bcl-2, Bcl-X_L_, TRAIL-R1/DR4, TRAIL-R2/DR5 and β-actin in tumor tissues derived from control and/or treated on week 6 was measured by the Western blot analysis.

We confirmed the immunohistochemistry data by examining the expression of these proteins by the Western blot analysis (Fig. 3). TRAIL has no effect on the expression of Bcl-2 family members (Bax, Bak, Bcl-2, and Bcl-X_L_) and death receptors (DR4 and DR5). By comparison, curcumin induced the expression of Bak, Bax, DR4 and DR5, and inhibited the expression of Bcl-2 and Bcl-X_L_. These data are in agreement with immunohistochemistry data where the expression of proapoptotic Bak, Bax, DR4 and DR5 proteins were induced and the expression of antiapoptotic Bcl-2 and Bcl-X_L _proteins were inhibited.

### *In vivo *regulation of cell cycle inhibitors (p21^/WAF1/CIP1 ^and p27^/KIP1^) and cyclin D1 by curcumin and/or TRAIL

**Figure 4:**
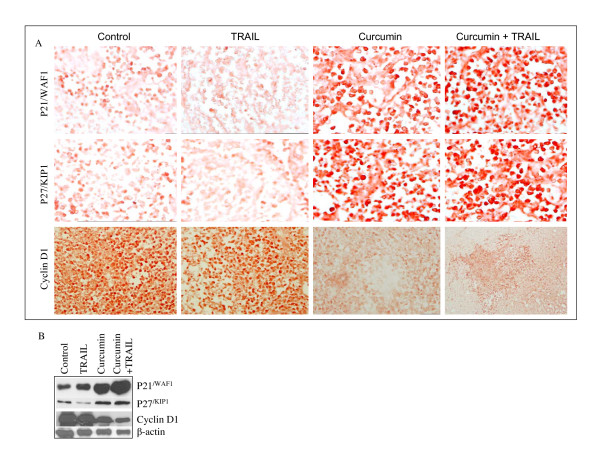
Effects of curcumin and/TRAIL on cell cycle inhibitors and cyclin D1. (A), Immunohistochemistry was performed to measure the expression of cell cycle inhibitors (p21^/WAF1 ^and p27^/KIP1^) and cyclin D1 in tumor tissues derived from control and/or treated mice on week 6. (B), Expression of p21^/WAF1^, p27^/KIP1^, cyclin D1 and β-actin in tumor tissues derived on week 6 were measured by the Western blot analysis.

Since curcumin caused growth arrest by inducing expression of cell cycle inhibitors p21^/WAF1/CIP1 ^and p27^/KIP1 ^*in vitro*, we sought to validate this phenomenon *in vivo *in xenografted tumors. Treatment of mice with curcumin resulted in the induction of cell cycle inhibitors p21^/WAF1/CIP1 ^and p27^/KIP1 ^and inhibition of cyclin D1 (Fig. 4A). By comparison, TRAIL had no effect on the expression of p21, p27 and cyclin D1 compared to control group. However, the combination of curcumin and TRAIL showed upregulation of p21 and p27, and inhibition of cyclin D1 compared to that of curcumin or TRAIL treated group. We next confirmed the immunohistochemistry data by examining the expression of these proteins by the Western blot analysis (Fig. 4B). TRAIL has no significant effect on the expression of p21, p27 and cyclin D1. By comparison, curcumin induced the expression of p21 and p27, and inhibited the expression of cyclin D1. These mice data are in agreement with *in vitro *data where curcumin upregulated the expression of p21 and p27, and inhibited the expression of cyclin D1 [35].

### *In vivo *regulation of NFκB, Cox-2 and IL-8 by curcumin and/or TRAIL

**Figure 5:**
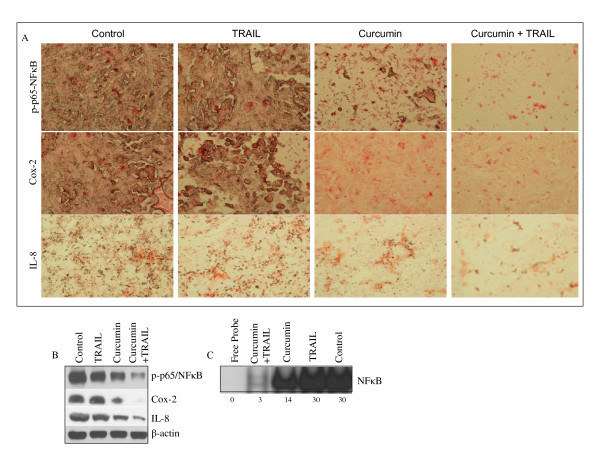
Effects of curcumin and/TRAIL on markers of phospho-p65NFκB, Cox-2, and IL-8. (A), Immunohistochemistry was performed to measure the expression of phospho-p65NFκB, Cox-2 and IL-8 in tumor tissues derived from control and/or treated mice on week 6. (B), Expression of phospho-p65NFκB, Cox-2, IL-8 and β-actin in tumor tissues derived on week 6 were measured by the Western blot analysis. (C), NFκB-DNA binding activity. Nuclear extracts were prepared from tumor tissues derived from different treatment groups on week 6. NFκB-DNA binding activity was measured by Gelshift assay as described in Materials and Methods. The relative nuclear NFκB-DNA binding activities were quantified by scanning densitometry.

The NFκB family of transcription factors has been shown to be constitutively activated in cancer cells and thus regulates genes involved in cell proliferation, apoptosis, metastasis and angiogenesis [36]. Since NFκB, Cox-2 and IL-8 play major role in cancer cell proliferation, and immune response, we sought to examine the effects of curcumin and/or TRAIL on the expression of these proteins by immunohistochemistry in tumor tissues derived from xenograft experiment (Fig. 5A). While TRAIL was ineffective, treatment of xenogrfated mice with curcumin resulted in inhibition of NFκB activation (as measured by phospho-p65 antibody), and cox-2 and IL-8 expression than control group. Furthermore, the combination of curcumin and TRAIL was more effective than curcumin alone in inhibiting NFκB activation and expression of cox-2 and IL-8.

We next confirmed the immunohistochemistry data by examining the expression of these proteins by the Western blot analysis (Fig. 5B). TRAIL has no significant effect on the phosphorylation of p-p65, and expression of cox-2 and IL-8. By comparison, curcumin inhibited the phosphorylation of p-p65, and expression of Cox-2 and IL-8. These data suggest that curcumin can inhibit NFκB activation and its gene products such as Cox-2 and IL-8.

Since curcumin inhibited the phosphorylation of p65 subunit of NFκB, we next measured the NFκB-DNA binding activity in tumor tissues derived from control, TRAIL, curcumin, and curcumin plus TRAIL treated groups. Nuclear extracts were prepared from tumor tissues derived from different treatment groups, and NFκB-DNA binding activity was measured by gelshift assay (Fig. 5C). TRAIL had no effect on NFκB-DNA binding activity compared to that of untreated mice. By comparison, curcumin inhibited NFκB-DNA binding activity by 53%. Interestingly, the combination of curcumin and TRAIL had more inhibitory effects on NFκB-DNA binding activity than curcumin alone. These data suggest that the inhibition of NFκB transcription factor by curcumin may play a major role in inducing sensitivity of TRAIL-resistant cells.

### *In vivo *regulation of angiogenesis by curcumin and/or TRAIL

**Figure 6:**
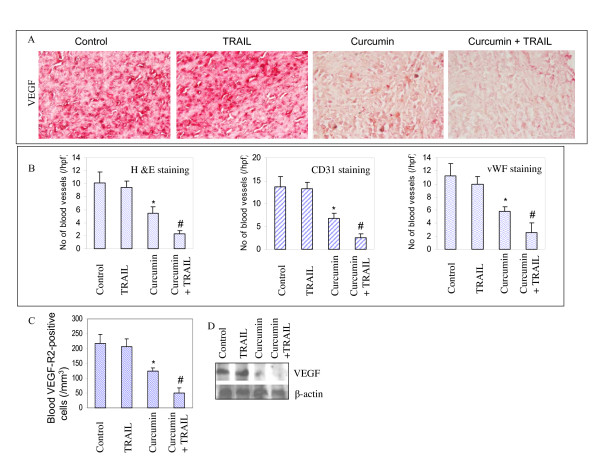
Effects of curcumin and/TRAIL on markers of angiogenesis. (A), Immunohistochemistry was performed to measure the expression of VEGF in tumor tissues derived from control and treated mice on week 6. (B), Left panel, tumor tissue sections derived from control and treated mice on week 6 were stained with H & E and the number of blood vessels in ten field at 400 × magnification were counted. Each column represents the mean ± SD. * or # = significantly different from control, P < 0.05. Middle panel, blood vessel quantification in tumors derived on week 6. Tumor sections from control and treated mice were stained with anti-CD31 antibody, and the number of CD31-positive blood vessels was counted. The results are shown as the mean ± SD. * or # = significantly different from control, P < 0.05. Right panel, tumor sections from control and treated mice obtained on week 6 were stained with anti-von Willebrand Factor (vWF) antibody to evaluate blood vessels. The results are shown as the mean ± SD. (C), VEGF receptor 2 (VEGF-R2)-positive circulating endothelial cells in mice on week 6. The blood cells from peripheral blood attached to the slide were stained with anti-VEGF-R2 antibody, and the number of positive cells was counted under a microscope. The results are shown as the mean ± SD. * or # = significantly different from control, P < 0.05. (D), Expression of VEGF and β-actin in tumor tissues derived on week 6 were measured by the Western blot analysis.

Whether regression in tumor growth by curcumin was due to inhibition of angiogenesis, we analyzed the markers of angiogenesis by immunohistochemistry in tumor samples. Examination of tumor tissues by immunohistochemistry showed that control mice had increased VEGF-positive endothelial cells compared to curcumin treated mice (Fig. 6A). TRAIL had no effect on VEGF staining. The combination of curcumin and TRAIL showed significantly less VEGF staining than that noted in tumors from mice treated with either agent alone.

We next examined the effects of curcumin and/or TRAIL treatment on number of blood vessels in tumor tissues by utilizing three different approaches (Fig. 6B). Blood vessels were examined by staining the tumor tissues by H&E, anti-CD31 antibody, and anti-vWF antibody. TRAIL treatment had no effect on number of blood vessels. By comparison, treatment of mice with curcumin caused an inhibition in number of blood vessels. The combination of curcumin with TRAIL further inhibited the number of blood vessels.

Several laboratories, including ours, have demonstrated that numbers of circulating vascular endothelial growth factor receptor 2 (VEGF-R2)-positive endothelial cells correlate directly with increase in tumor angiogenesis and can serve as *in vivo *indicators of tumor angiogenesis [27,37,38]. As expected, control mice had increased circulating VEGF-R2-positive endothelial cells compared to curcumin treated mice (Fig. 6C). Curcumin plus TRAIL-treated group had more inhibitory effects on VEGFR2-positive cells than that of curcumin or TRAIL treated group. Thus, these data strongly demonstrate that curcumin can inhibit tumor growth by inhibiting angiogenesis, and may also sensitize TRAIL-resistant tumor cells *in vivo*.

We next confirmed the immunohistochemistry data of VEGF expression by examining the protein levels by the Western blot analysis (Fig. 6D). TRAIL has no significant effect on VEGF expression. By comparison, curcumin or curcumin plus TRAIL inhibited the expression of VEGF.

### *In vivo *regulation of metastasis by curcumin and/or TRAIL

**Figure 7:**
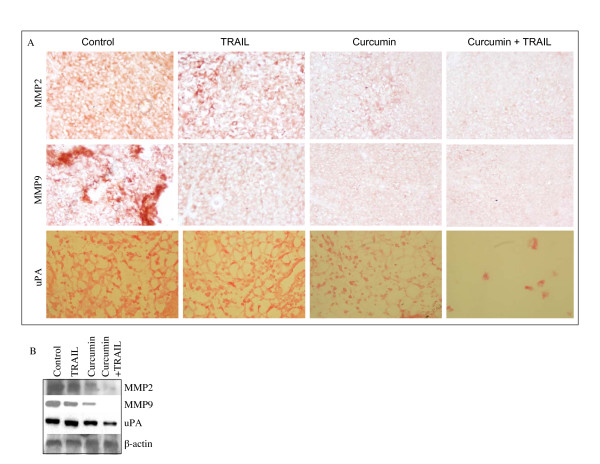
Effects of curcumin and/TRAIL on markers of metastasis. (A), Immunohistochemistry was performed to measure the expression of MMPs (MMP-2 and MMP-9) and uPA in tumor tissues derived from control and treated mice on week 6. (B), Expression of MMP-2, MMP-9, uPA and β-actin in tumor tissues derived on week 6 were measured by the Western blot analysis.

Elevated expression of matrix metalloproteinases (MMPs) and uPA are associated with increased metastatic potential in many tumor cells [39-42]. We therefore sought to examine the effects of curcumin on MMP-2, MMP-9, and uPA on tumor tissues derived from xenografted nude mice. Treatment of xenogrfated mice with curcumin resulted in inhibition of MMP-2, MMP-7, and uPA expression than those of control or TRAIL group (Fig. 7A). The combination of curcumin and TRAIL was more effective in inhibiting MMP-2, MMP-7, and uPA expression than single agent alone. We next confirmed the immunohistochemistry data by examining the expression of these proteins by the Western blot analysis (Fig. 7B). TRAIL has no significant effect on the expression of MMP-2, MMP-9 and uPA. By comparison, curcumin or curcumin plus TRAIL inhibited the expression of MMP-2, MMP-9 or uPA. These data suggest that curcumin can inhibit prostate cancer progression by inhibiting metastasis.

## Discussion

We have recently shown that curcumin induces apoptosis in TRAIL-sensitive PC-3 cells, and sensitizes TRAIL-resistant LNCaP cells *in vitro *through activation of multiple signaling pathways [35]. Curcumin-induced apoptosis engages mitochondria, which was evident by drop in mitochondrial membrane potential and activation of caspase-3 and caspase-9 in both prostate cancer PC-3 and LNCaP cells [35]. Curcumin induced expression of proapoptotic proteins (Bax, Bak, PUMA, Noxa and Bim), death receptors (TRAIL-R1/DR4 and TRAIL-R2/DR5), and inhibited expression of antiapoptotic proteins (Bcl-2 and Bcl-X_L_) and IAPs (XIAP and survivin) [35]. Since these proteins regulate cell-intrinsic and/or cell-extrinsic pathways of apoptosis, and they may be responsible for sensitization of TRAIL-resistant LNCaP cells. In the present study, we have demonstrated that curcumin inhibited the growth of LNCaP xenografts, metastasis and angiogenesis. Although the TRAIL was ineffective alone, the combination of curcumin and TRAIL had greater effect on tumor growth inhibition, metastasis and angiogenesis than curcumin.

*In vitro *curcumin downregulated the expression of Bcl-2, and Bcl-X_L _and upregulated the expression of p53, Bax, Bak, PUMA, Noxa, and Bim at mRNA and protein levels in prostate cancer cells [14]. We have also demonstrated that curcumin upregulated the expression, phosphorylation, and acetylation of p53 in androgen-dependent LNCaP cells [14]. The ability of curcumin to regulate gene transcription was also evident as it caused acetylation of histone H3 and H4 in LNCaP cells [14]. Furthermore, treatment of LNCaP cells with curcumin resulted in translocation of Bax and p53 to mitochondria, production of reactive oxygen species, drop in mitochondrial membrane potential, release of mitochondrial proteins (cytochrome c, Smac/DIABLO and Omi/HtrA2), and activation of caspase-3 leading to apoptosis [14]. Furthermore, deletion of Bax and Bak genes completely inhibited curcumin-induced cytochrome c and Smac/DIABLO release in mouse embryonic fibroblasts [13]. In the present study, tumor tissues derived from curcumin treated mice showed that curcumin inhibited the exprerssion of Bcl-2 and Bcl-X_L_, and induced the expression of Bax and Bak. The combinatioin of curcumin and TRAIL was more effective in regulating Bcl-2 family members than single agent alone. Our *in vitro *and *in vivo *studies demonstrate that curcumin can engage cell-intrinsic pathway of apoptosis by regulating the expression of Bcl-2 family of proteins.

We and others have recently shown that curcumin caused a growth arrest at G1/S stage in several cancers including prostate [43-45]. The G1/S phase arrest by curcumin was associated with the induction of p21^/WAF1^, p27^/KIP1^, and p16, and inhibition of cyclin D1, cyclin E, Cdk4 and cdk 6 [45]. The ability of curcumin to induce cdk inhibitors p21^/CIP1 ^and p27^/KIP1 ^and inhibit cyclin D1 expression was also confirmed in our xenograft experiment. In a recent study, we have demonstrated that inhibition of p21^/CIP1 ^inhibited curcumin-induced cell cycle arrest and apoptosis [46]. We and others have also demonstrated that curcumin induces the degradation of cyclin E expression through ubiquitin-dependent pathway in several cancer cell lines [44,45]. Interestingly, deregulated expression of cyclin E correlated with chromosome instability [47], malignant trasformation [48], tumor progression [49], and patient survival [50]. Overall, our data suggest that curcumin induces growth arrest at G1/S stage of cell cycle.

Entry of malignant cells into the vasculature (i.e. intravasation) requires proteolytic remodeling of the extracellular matrix so that tumor cells may pass through the local stroma and penetrate the vessel wall. The circulatory system then provides a means of transporting tumor cells to distant sites where they extravasate and establish metastatic lesions. Matrix metalloproteinase (MMP) is up-regulated in many tumor types and has been implicated in tumor progression and metastasis. MMP is critical for pericellular degradation of the extracellular matrix, thereby promoting tumor cell invasion and dissemination. To grow efficiently *in vivo*, tumor cells induce angiogenesis in both primary solid tumors and metastatic foci. Our results showed that curcumin significantly inhibited the growth of TRAIL-resistant LNCaP xenografts and sensitized these xenografts to undergo apoptosis by TRAIL. Tumor tissues derived from curcumin treated mice showed that curcumin inhibibited proliferation (PCNA and Ki67 staining), induced apoptosis (TUNEL staining), metastasis (uPA, MMP-2 and MMP-9 staining), and angiogenesis (CD31 and VEGF staining). Curcumin also inhibited VEGFR2-positive circulating endothelial cells. Treatment of LNCaP xenografted mice with TRAIL alone had no effect on tumor growth, apoptosis, metastasis and angiogenesis. Our recent *in vitro *studies demonstrated that curcumin inhibits capillary tube formation and endothelial cell migration, and the inhibitory effects of curcumin were enhanced in the presence of ERK MAP kinase inhibitor [35]. These data suggest that curcumin can inhibit tumor growth by inhibiting apoptosis, metastasis and angiogenesis.

TRAIL induces apoptosis in cancer cells which express TRAIL-R1/DR4 and TRAIL-R2/DR5. We have shown that the upregulation of death receptors by chemotherapeutic drugs, irradiation and chemopreventive agents enhance or sensitize cancer cells to TRAIL treatment [28,30,35,51-58]. Specifically, TRAIL-resistant LNCaP cells can be sensitized by chemotherapeutic drugs and irradiation *in vitro *and *in vivo *through upregulation of death receptors DR4 and/or DR5 [27,28]. Similarly, our *in vitro *study has demonstrated the upregulation of DR4 and DR5 in PC-3 and LNCaP cells by curcumin [35]. Interestigly, curcumin sensitized TRAIL-resistant LNCaP xenografts by inhibiting tumor cell proliferation and inducing apoptosis which were correlated with induction of death receptors DR4 and DR5. Death receptor (DR4 and/or DR5) regulation has been shown to be under the control of transcription factor NFκB, SP1 and p53 [59-64]. Inducible silencing of KILLER/DR5 *in vivo *promoted bioluminescent colon tumor xenograft growth and confers resistance to chemotherapeutic agent 5-fluorouracil [65]. These finding suggest that upregulation of death receptors DR4 and DR5 by curcumin may be one of the mechanisms by which curcumin enhances the therapeutic potnetial of TRAIL.

The NFκB family of transcription factors has been shown to be constitutively activated in various human malignancies, including a number of solid tumors, leukemias, and lymphomas [66]. NFκB is shown to contribute to development and/or progression of malignancy by regulating the expression of genes involved in cell growth, differentiation, apoptosis, angiogenesis and metastasis [66]. Prostate cancer cells have been reported to have constitutive NFκB activity due to increased activity of the IκB kinase complex [67]. Furthermore, an inverse correlation between androgen receptor (AR) status and NFκB activity was observed in prostate cancer cell lines [68]. In prostate cancer cells, NFκB may promote cell growth and proliferation by regulating expression of genes such as c-myc, cyclin D1, and IL-6 [66,69], and inhibit apoptosis through activation of expression of anti-apoptotic genes, such as Bcl-2 and Bcl-X_L_. NFκB-mediated expression of genes, involved in angiogenesis (IL-8, VEGF), invasion and metastasis (MMP-9, uPA, uPA receptor), may further contribute to the progression of prostate cancer. Constitutive NFκB activity has also been demonstrated in primary prostate cancer tissue samples and suggested to have prognostic importance for a subset of primary tumors. In the present study, curcumin inhibited the activation of NFκB and its gene products such as VEGF, Bcl-2, Bcl-X_L_, uPA, cyclin D1, MMP-2, MMP-9, COX-2 and IL-8 in LNCaP xenografted tumors. These findings suggest that NFκB may play a role in human prostate cancer development, and/or progression, and curcumin can inhibit these processes through regulation of NFκB-regulated gene products.

## Conclusion

Our *in vivo *experiments have demonstrated that curcumin sensitizes TRAIL-resistant LNCaP cells through multiple mechanisms. It induces death receptors, upregulates proapoptotic members of Bcl-2 family (Bax and Bak), inhibits antiapoptotic Bcl-2 proteins (Bcl-2 and Bcl-X_L_) and markers of cell proliferation (PCNA and Ki67), and induces expression of cell cycle inhibitors p21^/CIP1^, and p27^/KIP1^. Furthermore, curcumin can also inhibit the activation of NFκB and its gene products (e.g. VEGF, Bcl-2, Bcl-X_L_, uPA, cyclin D1, MMP-2, MMP-9, COX-2 and IL-8) *in vitro *[35], and *in vivo *(current study), which play significant roles in invasion, metastasis and angiogenesis. All these events will significantly contribute to the antiproliferative and antitumor activities of curcumin. Clinical trials on curcumin have demonstrated that (1) curcuma extract can be administered safely to patients at doses of up to 2.2 g daily, equivalent to 180 mg of curcumin; and (2) curcumin has low oral bioavailability in humans and may undergo intestinal metabolism [70]. Our studies posses strong clinical potential because curcumin either alone or in combination with TRAIL can be used to prevent and/or treat prostate cancer.

## Methods

### Reagents

Antibodies against CD31, VEGF, VEGFR2, Bcl-2, Bcl-X_L_, Bax, Bak, TRAIL-R1/DR4, TRAIL-R2/DR5 and β-actin were purchased from Santa Cruz Biotechnology Inc. (Santa Cruz, CA). Antibodies against p21, p27, phospho-p65-NFκB, Cox-2, IL-8, cyclin D1, uPA, MMP-2, and MMP-9 were purchased from Cell Signaling Technology, Inc. (Danvers, MA). Enhanced chemiluminescence (ECL) Western blot detection reagents were from Amersham Life Sciences Inc. (Arlington Heights, IL). Terminal Deoxynucleotidyl Transferase Biotin-dUTP Nick End Labeling (TUNEL) assay kit was purchased from EMD Biosciences/Calbiochem (San Diego, CA). TRAIL was purified as described elsewhere [71]. Curcumin was purchased from LKT Laboratories, Inc. (St. Paul, MN).

### Western blot analysis

Western blot analysis was performed as we described earlier [13]. Protein bands were visualized on X-ray film using an enhanced chemiluminescence system.

### Xenograft assays in nude mice

Athymic nude mice (Balb c nu/nu, 4–6 weeks old) were purchased from the National Cancer Institute (Frederick, MD). LNCaP cells (2 × 10^6^cells as a 50% suspension in matrigel, Becton Dickinson, Bedford, MA) in a final volume of 0.1 ml were injected subcutaneously at right flank of Balb c nude mice. When the average tumor volume reached about 100 mm^3^, mice were randomized into four groups of 10 mice/group, and the following treatment protocol was implemented: *Group 1*, vehicle control (0.1 ml normal saline containing 0.5 % DMSO) administered by oral injection, three times/week (Monday, Wednesday and Friday) beginning the day of tumor cell implantation through out the duration of experiment; *Group 2*, TRAIL (15 mg/kg) administered i.v. on day 1, 7, 14, and 21; *Group 3*, curcumin (30 mg/kg, in 0.1 ml normal saline containing 0.5 % DMSO) administered by oral injection, three times/week (Monday, Wednesday and Friday) beginning the day of tumor cell implantation through out the duration of experiment; *Group 4*, curcumin and TRAIL, curcumin administered through oral injection, and TRAIL administered i.v. Mice were housed under pathogen-free conditions and maintained on a 12 h light/12 h dark cycle, with food and water supplied *ad libitum*. Tumor volume was calculated using the equation: (volume = length × width × depth × 0.5236 mm^3^). The *in vivo *experiment was performed under IACUC's approved protocol.

### Immunohistochemistry

Immunohistochemistry was performed as described earlier (28, 29). In brief, tumor tissues were collected on week 6, excised and fixed with 10% formalin, embedded in paraffin and sectioned. Tissue sections were stained with primary antibodies against Bax, Bak, Bcl-2, Bcl-X_L_, DR4, DR5, Ki-67, PCNA, p21/^WAF1/CIP1^, p27^/Kip1^, IL-8, Cox-2, phospho-p65-NFkB, CD31, VEGF, VEGFR2, MMP-2, MMP-9 and uPA or TUNEL reaction mixture. For immunohistochemistry, sections were fixed in cold 100% acetone for 3 min, air-dried, and incubated with various primary antibodies at room temperature for 4 h. Subsequently, slides were washed three times in PBS and incubated with secondary antibody at room temperature for 1 h. Finally, alkaline phosphatase or hydrogen peroxide polymer-AEC chromagen substrate kits were used as per manufacturer' instructions (Lab Vision Corporation). After washing with PBS, Vectashield (Vector Laboratories) mounting medium was applied and sections were coverslipped and imaged.

### Electrophoretic mobility shift assay

EMSA was performed as we described elsewhere [59].

### Statistical analysis

The mean and SD were calculated for each experimental group. Differences between groups were analyzed by one or two way ANOVA. The non-parametric Mann-Whitney U test was performed to assess the difference of tumor volume between control and treatment group. To assess the difference between two groups under multiple conditions, one-way ANOWA followed by Bonferoni's multiple comparison tests were performed using PRISM statistical analysis software (GrafPad Software, Inc., San Diego, CA). Significant differences among groups were calculated at P < 0.05.

## Competing interests

The author(s) declare that they have no competing interests.

## Authors' contributions

SS, SG, and QC have performed the experiments and drafted the manuscript. RS has directed the project and edited the manuscript. All authors read and approved the final manuscript.
